# Surgery

**Published:** 1915-03

**Authors:** C. A. Moore


REFERENCES.

1 *Am. J. M. Sc.*, 1914, cxlviii. 33.
2 *Bull. Johns Hopkins Hosp.*, 1914, xxv. 133.
3 *Med. Rec.*, 1915, lxxxvii. 127.
4 *Brit. M. J.*, 1913, i. 1,257.
5 *Ibid.*, 1914, i. 1,393.

### SURGERY.

**Sarcoma of the long bones.**—This interesting subject has recently been reviewed by Coley[1] in a communication of great value. The illuminating discussion at the Royal Society of Medicine in November, 1912, furnished him with some interesting statistics, but the greater part of the paper is founded on the author's personal experience.

He refers to the gloomy statistics brought forward by Gask at the discussion referred to—that, out of twenty-five cases of

1 *Ann. Surg.*, lx. 537, *et seq.*

periosteal sarcoma of the femur, not one was alive at the end of three years, and only one had failed to recur at the end of eighteen months. In eight humerus cases, all ended fatally within the three-year limit. Of the twenty-eight St. Thomas's Hospital cases, all were dead within the same time.

The dismal fact is apparent, that, in spite of new aids to early and accurate diagnosis, the results are no improvement on those of Butlin of a generation ago, the ultimate prognosis in cases of periosteal sarcoma being still practically hopeless.

Coley claims for his cases considerably improved results, giving as his reason, not earlier diagnosis—for many of the cases were inoperable when first seen by him—but the use of his mixture of the toxins of Bacillus Prodigiosus and Streptococcus Erysipelatis.

The author protests against the traditional treatment (possibly one might say that "obsolescent" would be a fairer word to use than "traditional") of all cases of sarcoma of the long bones by immediate amputation. He holds that every case should be subjected to a two or three weeks' course of injection of the mixed toxins, and only in the event of no improvement manifesting itself should operation be undertaken. To the obvious objection to this plan, that to wait two or three weeks to see the effect, if any, of toxin treatment may be waste of valuable time, he replies that in any case which cannot wait three weeks, amputation is a hopeless proceeding and scarcely worth undertaking.

On the other hand, in a proportion of cases—admittedly probably a small one—the limb will be saved by the toxin treatment, and, in any case, it will enhance the prospects of final success in the shape of freedom from recurrence after amputation or other operative procedure.

The author gives details of twenty-seven cases successfully treated. In eleven the limb was saved. Many cases did not react to the toxin treatment, therefore amputation was undertaken, followed by a further course of injections, and no recurrence followed.

Space forbids mention of the many interesting cases cited, and one striking instance must suffice. The case was one of a large periosteal sarcoma of the femur in a man of 19 years, with widespread metastases in pectoral and ilio-lumbar regions. Hip-joint amputation had been advised and refused. After a two months' course of injections, all trace of the diease disappeared, and the patient remained well for ten years. He then developed in the skin of the thigh at the site of an old X-ray burn a very highly malignant tumour, "epithelioma and sarcoma combined," which proved fatal.

As to diagnosis, the main subjective symptom is deep-seated, boring pain, worse at night or after use of the limb. When, later, a palpable tumour develops, local heat is often present, and the patient's temperature may rise to 101–102°.

Reference is made to the theory, originated by Eve, that the difference in nature of myeloma and sarcoma depends on the site of the disease rather than on the structure of the tumour—the nearer the trunk the greater the malignancy is true of myeloma as well as of periosteal sarcoma. Coley thinks this is by no means universal, and quotes one of the most malignant cases he has ever met with, a periosteal sarcoma of the meta carpus. He dissents also from the opinion, commonly held, that a myeloma is necessarily only locally malignant and not associated with metastatic deposits, giving cases to support his contention.

Differences in the course of such cases may be due in many instances to difficulties in making the pathological diagnosis, instances having often occurred in which pathologists of very great experience have been in error.

Going on to refer to the value of X-rays in diagnosis, Coley utters a warning that considerable experience in interpreting skiagrams is essential if errors are to be avoided. The X-rays, at best, are subject to limitations. For instance, they show little or no difference between a primary sarcoma of the shaft of a bone and a carcinoma secondary to disease elsewhere.

He utters a word of warning against the indiscriminate use of exploratory incision as a means of establishing the diagnosis, valuable as it is in suitable cases. It is liable to lead the surgeon and pathologist into serious error, owing to the incision not having been carried sufficiently deep, and thus not including a typical part of the tumour. Other obvious dangers are the risk of dissemination of the growth, and the liability of causing sinuses, which do not heal. Some situations are especially unsuitable for this method of investigation, notably the popliteal region of the femur. When the tumour is cut into, a certain opinion as to its malignancy can generally be given from naked-eye appearance alone.

The author quotes with approval Makins's opinion that no sarcoma of long bones should be cut into unless the surgeon is prepared to amputate, if necessary, within twenty-four hours.

In cases in which clinical and pathological evidence differs, Coley agrees with Corner that the clinical evidence is the more weighty. In many cases of error in pathological diagnosis the fault is the surgeon's, in not cutting deep enough, and so supplying the pathologist with a piece for section which is not typical. He goes on to refer to the varying malignancy of cases of chondrosarcoma, ranging as they do from the lowest to almost the highest. They should always be regarded with grave suspicion.

The whole paper is a most interesting and instructive one.

While most British surgeons are unable to endorse entirely Dr. Coley's enthusiasm for treatment by the toxin method, few will have failed to come across a case or cases in which it undoubtedly helped to save life or limb from the ravages of this terrible disease.

C. A. MOORE.

### DISEASES OF CHILDREN.

